# Early Growth Response Gene-1 Suppresses Foot-and-Mouth Disease Virus Replication by Enhancing Type I Interferon Pathway Signal Transduction

**DOI:** 10.3389/fmicb.2018.02326

**Published:** 2018-09-27

**Authors:** Zixiang Zhu, Xiaoli Du, Pengfei Li, Xiangle Zhang, Fan Yang, Weijun Cao, Hong Tian, Keshan Zhang, Xiangtao Liu, Haixue Zheng

**Affiliations:** State Key Laboratory of Veterinary Etiological Biology, National Foot and Mouth Diseases Reference Laboratory, Key Laboratory of Animal Virology of Ministry of Agriculture, Lanzhou Veterinary Research Institute, Chinese Academy of Agricultural Sciences, Lanzhou, China

**Keywords:** foot-and-mouth disease virus, early growth response gene-1, interferon, viral replication, antiviral response

## Abstract

Early growth response gene-1 (EGR1) is a multifunctional transcription factor that is implicated in viral infection. In this study, we observed that foot-and-mouth disease virus (FMDV) infection significantly triggered EGR1 expression. Overexpression of EGR1 suppressed FMDV replication in porcine cells, and knockdown of EGR1 considerably promoted FMDV replication. A previously reported FMDV mutant virus (with two amino acids mutations in SAP domain) that displays a strong type I interferon (IFN) induction activity was used in this study. We found that SAP mutant FMDV infection induced a higher expression of EGR1 than wildtype FMDV infection, and also triggered higher IFN-β and IFN-stimulated genes (ISGs) expression than wildtype FMDV infection. This implied a link between EGR1 and type I IFN signaling. Further study showed that overexpression of EGR1 resulted in Sendai virus (SeV)-induced IFN-stimulated response element (ISRE) and NF-κB promoter activation. In addition, the SeV-induced ISGs expression was impaired in EGR1 knockdown cells. EGR1 upregulation promoted type I IFN signaling activation and suppressed FMDV and Seneca Valley virus replication. Suppression of the transcriptional activity of EGR1 did not affect its antiviral effect against FMDV. This study reveals a new mechanism evolved by EGR1 to enhance type I IFN signaling and suppress FMDV replication.

## Introduction

Foot-and-mouth disease virus (FMDV) is a non-enveloped virus with positive-sense and single-stranded RNA genome. The viral genome is approximately 8.5 kb nucleotides in length, including a single large open reading frame that encodes a polyprotein. The polyprotein is subsequently processed by viral proteases during protein synthesis, generating several intermediates and 12 mature proteins (Sobrino and Domingo, 2001; Grubman and Baxt, 2004). During co-evolution with the hosts, these viral proteins have acquired many functions to counteract host antiviral responses, cause immunosuppression, and promote viral replication and infection (Mason et al., 2003; Rodriguez Pulido and Saiz, 2017). Therefore, FMDV causes an acute vesicular disease of infected animals, which is called foot-and-mouth disease (FMD). FMD is a highly contagious disease that can lead to significant economic losses to the local livestock industry (Rweyemamu et al., 2008a; Paton and Taylor, 2011; Zai-Xin, 2015; Bouguedour and Ripani, 2016). The understanding of host-FMDV interaction as well as the involved mechanism contributes to the planning of new strategies for FMD prevention (Domingo et al., 2005; Rweyemamu et al., 2008b; Rodriguez Pulido and Saiz, 2017). Accordingly, many researches on host responses in FMDV-infected cells have to be investigated.

Early growth response gene-1 (EGR1), also designated zif268, is a host transcriptional regulator that expresses rapidly after a number of stimuli like oxygen deprivation, growth factors, cytokines, shear stress and injury (Brand et al., 1992; Khachigian et al., 1997; Nishi et al., 2002; Jo et al., 2011; Li et al., 2013). EGR1 is involved in diverse biologic functions and a broad variety of host signal transduction cascades that mediates cell growth, survival, differentiation, apoptosis and proliferation (Pagel and Deindl, 2011; Papanikolaou et al., 2014). Different pathways have been identified that participate in EGR1 induction and then regulates several biological behaviors. Such as, the Ras homologue gene family (Rho) genes are involved in cell cycle progression, and the Rho/Rho-kinase pathway has been shown to regulate EGR1 expression (Barrientos et al., 2007; Pagel and Deindl, 2011). As a zinc-finger DNA-binding protein, EGR1 also regulates expression of diverse gene families by binding to promoter sequences of target genes (Papanikolaou et al., 2014). Therefore, EGR1 is involved in activation of signal transduction of many pathways.

Several studies indicate that EGR1 is linked to viral infection and immune response. EGR1 modulates pro-apoptotic pathway and promotes Venezuelan equine encephalitis virus (VEEV) replication (Baer et al., 2016). Knockdown of EGR1 in Rhabdomyosarcoma cells decreases enterovirus 71 (EV71) replication (Song et al., 2015). It seems that EGR1 might play a positive role in these viruses replication. However, EGR1 also appears critical for the initiation of immune response in B cells and T cells. EGR1 plays roles in regulation of the expression of sever cytokines including interleukin-2, CD44, ICAM-1 and tumor necrosis factor genes (Skerka et al., 1995; McMahon and Monroe, 1996; Shin et al., 2009; Cubero and Nieto, 2012).

An SAP domain [scaffold-attachment factor (SAF)-A/B, apoptotic chromatin-condensation inducer in the nucleus (ACINUS) and PIAS (protein inhibitor of activated signal transducer and activator of transcription) domain] previously was identified within the FMDV L^pro^ by de los Santos et al. (2009). Mutation of L^pro^ SAP domain promotes type I IFN signaling activation and decreases virus growth. In addition, animals inoculated with the FMDV SAP mutant display strong neutralizing antibody response and T cell response comparing with infection with wildtype FMDV (de los Santos et al., 2009; Diaz-San Segundo et al., 2012). In this study, a robust EGR1 upregulation was observed in both wildtype and SAP mutant FMDV-infected cells comparing with the mock-infected cells by viewing the protein abundance of EGR1. Therefore, we investigated the correlation between FMDV infection and EGR1, and determined the antiviral role of EGR1 against FMDV. SAP mutant FMDV infection induced a higher expression of EGR1 than wildtype FMDV infection. SAP mutant FMDV infection also triggered higher IFN-β and IFN-stimulated genes (ISGs) expression than wildtype FMDV infection. We also found that overexpression of EGR1 enhanced Sendai virus (SeV)-induced interferon (IFN)-stimulated response element (ISRE) activation. SeV-induced ISGs expression was impaired in EGR1 knockdown cells, which may serve as a link between upregulation of EGR1 and type I IFN signaling. Further study showed that EGR1 enhanced TBK1 phosphorylation during FMDV infection. It indicated that EGR1 upregulation promoted type I IFN signaling activation by enhancing TBK1 phosphorylation and resulted in decreased FMDV replication. This study reveals a link between EGR1 and innate immune response during FMDV infection.

## Materials and Methods

### Cell Lines, Viruses and Reagents

Porcine kidney PK-15 cells, human embryonic kidney 293T cells (HEK293T) cells described previously (Zhu et al., 2016) were maintained in Dulbecco’s modified Eagle’s medium supplemented with 10% heat-inactivated fetal bovine serum, 100 U/ml penicillin, and 100 μg/ml streptomycin sulfate. All the cells were cultured at 37°C under 5% CO2. Sendai virus (SeV), a model RNA virus widely used to activate type I IFN signaling in cells, was kindly provide by Hongbing Shu’s Laboratory (Wuhan University, China) (Zhou et al., 2014; Li D. et al., 2016). FMDV strain O/BY/CHA/2010 (GenBank number: JN998085) described previously was used for virus infection (Zheng et al., 2012).

Commercial antibodies used in this study include an anti-EGR1 mouse monoclonal antibody (Abcam, Cambridge, MA, United States), anti-TBK1 rabbit antibody from Cell Signaling Technology (CST) Inc. (Beverly, MA, United States), anti-phospho-TBK1 rabbit antibody (CST), anti-c-Myc mouse antibody (Santa Cruz Biotechnology, Santa Cruz, CA, United States) and anti-β-actin mouse antibody (Santa Cruz Biotechnology). Anti-IFN-β and anti-IFN-α antibodies (5000 NU/ml, PBL Biomedical Laboratories) and IgG isotype antibodies were used in the type I IFN-blocking experiments as previously described (Trottier et al., 2009). Anti-FMDV VP1 protein polyclonal antibody was previously produced in our laboratory (Zhu et al., 2016). Transfection reagents include OPTI-MEM medium and the Lipofectamine 2000 that were purchased from Invitrogen. Poly (I:C) was purchased from InvivoGen. IFN-β was purchased from PBL Biomedical Laboratories.

### Plasmids and Transfection

The full-length porcine EGR1 cDNA fragment was cloned into a pcDNA^TM^3.1/myc-His(-)A vector (Invitrogen) to construct a Myc-tagged EGR1 eukaryotic expressing plasmid (Myc-EGR1, including a C-terminal Myc tag). The constructed plasmid was analyzed and verified by DNA sequencing. A series of plasmids expressing HA-tagged type I IFN pathway-related proteins [including MDA5, RIG-I(CARD), VISA, TBK1, IRF3 and IRF7], and the IFN-β promoter luciferase reporter plasmids and control plasmid Renilla luciferase pRL-TK were kindly provided by Hongbing Shu’s Laboratory (Zhou et al., 2014; Li D. et al., 2016). The plasmids were transfected into cells using OPTI-MEM medium and the Lipofectamine 2000 (Invitrogen) reagent according to the manufacture’s protocol.

### Real-Time Quantitative PCR (qPCR)

TRIzol reagent (Invitrogen) was used to extract cellular or viral RNA following the instruction of the protocol. The first-strand cDNA was synthesized by reverse transcription reaction with the extracted RNAs as templates. Reverse transcription was performed with M-MLV reverse transcriptase (Invitrogen) and random hexamer primers (TaKaRa) according to the manufacturer’s recommendations. The quantification of the cDNA was performed by qPCR. The relative amounts of the synthesized cDNA was determined as an indicator of the target transcripts. qPCR was carried out using SYBR Premix Ex Taq (Takara) on a QuantStudio 5 Real-Time PCR instrument (Applied Biosystems) according to the manufacturer’s instructions. The glyceraldehyde-3-phosphate dehydrogenase (GAPDH) gene was used for normalization in qPCR analysis. Relative transcript levels were calculated using 2^−ΔΔCT^ method as described previously (Zhu et al., 2016). All the primers used in this study were listed in **Table 1**.

**Table 1:** The primers used in this study.

Gene	Primers(5′→3′)	Application
Porcine-EGR1	Forward: TTATCTCGAGATGGCGGCAGCCAA	RT-PCR
	Reverse: GCAAGCTTGCAGATTTCAATTGTCC TGGGAGA	
FMDV	Forward: CACTGGTGACAGGCTAAGG	qPCR
	Reverse: CCCTTCTCAGATTCCGAGT	
P/H-GAPDH	Forward: ACATGGCCTCCAAGGAGTAAGA	qPCR
	Reverse: GATCGAGTTGGGGCTGTGACT	
P/H-EGR1	Forward: GACCACCTCACCACCCACAT	qPCR
	Reverse: CCGCAAGTGGATCTTGGTAT	
Porcine-IFN-β	Forward: GCTAACAAGTGCATCCTCCAAA	qPCR
	Reverse: AGCACATCATAGCTCATGGAAAGA	
Porcine-MX1	Forward: GAGGTGGACCCCGAAGGA	qPCR
	Reverse: CACCAGATCCGGCTTCGT	
Porcine-ISG15	Forward: GATCGGTGTGCCTGCCTTC	qPCR
	Reverse: CGTTGCTGCGACCCTTGT	
Human-IFN-β	Forward: GACATCCCTGAGGAGATTAAG	qPCR
	Reverse: ATGTTCTGGAGCATCTCATAG	
Human-ISG15	Forward: TGGACAAATGCGACGAACC	qPCR
	Reverse: CCCGCTCACTTGCTGCTT	
Human-MX1	Forward: ACCTCGTGTTCCAACTGAAG	qPCR
	Reverse: GTGTGATGAGCTCGCTGGTA	

### Immunoblotting Analysis

For Western blotting, the cells were collected at the indicated time points and. The lysed cell extracts were resolved by 10% SDS-PAGE and transferred onto a nitrocellulose membrane (Pall). The nitrocellulose membrane was then blocked with 10% skim milk powder in TBST (20 mM Tris, 55 mM NaCl, 0.1% Tween 20) overnight at 4°C. The membrane was incubated with primary and secondary antibodies as described previously (Zhu et al., 2013). The membrane was washed 3 × 5 min, before being protein abundance analysis. The antibody–antigen complexes were visualized using enhanced chemiluminescence detection reagents (Thermo).

### RNA Interference (RNAi)

Small interfering RNA (siRNA) was used to knockdown EGR1 protein expression. siRNA fragments were chemically synthesized by genepharma company (China). The sequences of the siRNAs used in this study include: 5′-CCAUGGACAACUACCCUAATT-3′ (EGR siRNA-353), 5′-GCCUAGUGAGCAUGACCAATT-3′ (EGR siRNA-749), and 5′-GCUGUCACCAACUCCUUCATT-3′ (EGR siRNA-1819). A non-targeting siRNA (NC siRNA) was used as a negative control. siRNA fragments were transfected into cells using Lipofectamine 2000 as described previously (Li W. et al., 2016). Forty-eight hours after siRNA transfection, cells were used for further experiments. To examine the effect of siRNA on EGR1 expression, EGR1 mRNA and protein abundance were measured by qPCR and Western blotting respectively.

### Dual Luciferase Reporter Assays

HEK293T cells seeded on 24-well plates were co-transfected with 100 ng luciferase reporter plasmid with 10 ng internal control Renilla luciferase reporter plasmid (to normalize for transfection efficiency) PRL-TK (Promega), together with the indicated plasmids and/or empty vector controls using Lipofectamine 2000 according to the manufacture’s instruction. To make the cells receive the same amounts of total plasmids, the empty vector plasmids were used in all transfection experiments. As for SeV-mediated type I IFN signaling pathway activation, the cells were mock-infected or infected with SeV (100HAU/mL) for 16 h; and the dual luciferase assays were then performed according to the Promega Dual-Luciferase Reporter Assay System protocol. The relative luciferase activity was expressed as arbitrary units by normalizing firefly to Renilla luciferase activity. As for type I IFN pathway adaptor molecules-induced IFN-element activation assay, the HEK293T cells were co-transfected with the reporter plasmids with the indicated plasmid or vector plasmid for 24 h; and the luciferase activities were measured.

### Statistical Methods

All experiments were performed at least in triplicate. The measured values are represented as mean ± SD from three independent experiments. The statistical significance analyses were performed using the Student’s *t*-test. Data considered significant when ^∗^*P* < 0.05, and highly significant when ^∗∗^*P* < 0.01.

## Results

### FMDV Infection Upregulates EGR1 Expression

**FIGURE 1:**
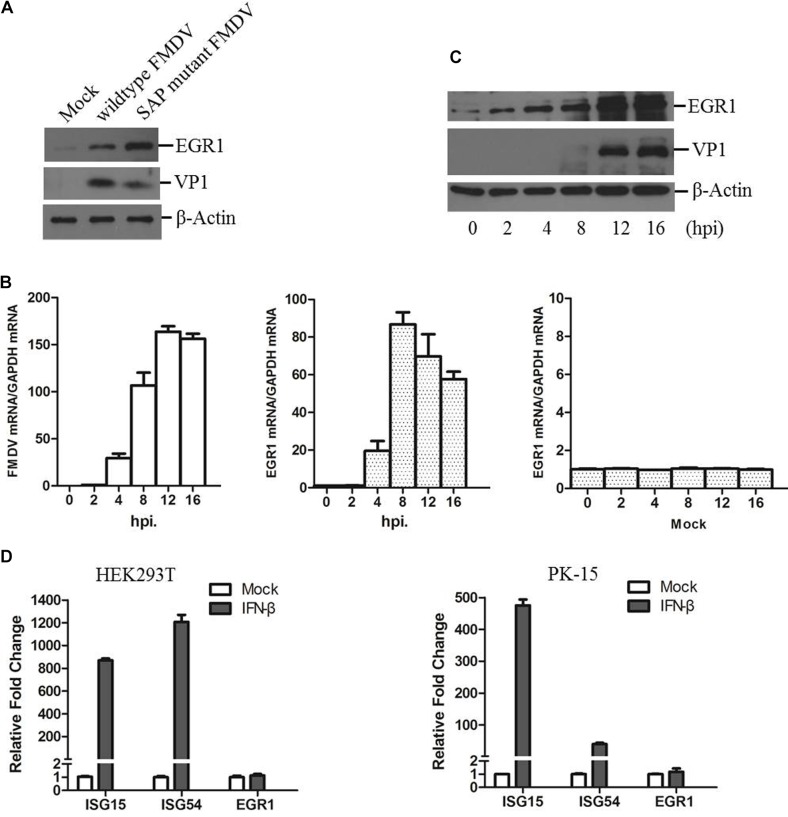
State of EGR1 in FMDV-infected cells. **(A)** PK-15 cells were incubated with equal amounts of SAP mutant FMDV or wildtype FMDV for 12 h, the abundance of EGR1 and viral VP1 protein was detected. **(B)** PK-15 cells were infected with wildtype FMDV or mock-infected for 0, 2, 4, 8, 12, or 16 h. The transcripts of EGR1 and viral RNA were detected by qPCR. **(C)** PK-15 cells were infected with wildtype FMDV for 0, 2, 4, 8, 12, or 16 h. The expression levels of EGR1 and VP1 protein were detected by Western blotting. **(D)** HEK293T or PK-15 cells were mock-treated or incubated with IFN-β at a concentration of 10 ng/ml for 12 h. The expression levels of ISG15, ISG54 and EGR1 was measured by qPCR.

PK-15 cells were infected by equal amounts of wildtype or SAP mutant FMDV for 12 h as previously described (Zhu et al., 2015). The expression levels of EGR1 and viral VP1 protein were detected by Western blotting. We observed that EGR1 protein level is significantly upregulated both in wildtype and SAP mutant FMDV-infected cells at 12 h postinfection (hpi) (**Figure 1A**). Therefore, the correlation between FMDV infection and EGR1 was further investigated. The dynamics of EGR1 in FMDV-infected cells were determined. Transcripts of EGR1 were considerably upregulated after FMDV infection and reached to the highest level at 8 hpi. No significant changes were observed in mock-infected cells (**Figure 1B**). EGR1 protein expression was also gradually upregulated as the infection progressed (**Figure 1C**). This indicates that FMDV infection triggers upregulation of EGR1. To investigate whether EGR1 is an IFN inducible gene, HEK293T and PK-15 cells were incubated with IFN-β to induce the expression of IFN inducible genes. The expression of two IFN inducible genes ISG15 and ISG54 was highly induced by incubation of IFN-β. However, the expression of EGR1 was not changed by treatment of IFN-β (**Figure 1D**). This indicated that EGR1 expression was not induced by IFN-β treatment; however, FMDV infection could induce EGR1 expression.

### EGR1 Plays an Anti-viral Role During FMDV Replication

**FIGURE 2:**
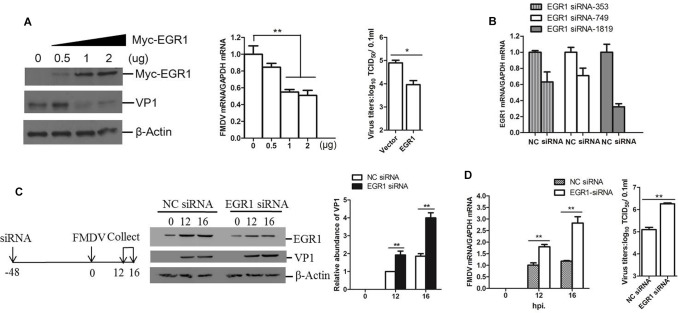
Antiviral effect of EGR1 against FMDV. **(A)** PK-15 cells were transfected with 0, 0.5, 1, or 2 μg of Myc-EGR1, the empty vector plasmids were used in all transfection experiments to ensure the cells receive the same amounts of plasmids. The cells were infected with 0.5 MOI of FMDV at 24 hpt and incubated for another 12 h. The viral protein and RNA were detected. The viral titers in the vector and EGR1 plasmids (2 μg) transfected cells were measured by TCID_50_ assay.**(B)** PK-15 cells were transfected with NC (negative control) or siRNA (EGR1 siRNA-353, EGR1 siRNA-749 or EGR1 siRNA-1819) for 48 h. The EGR1 mRNA levels were measured by qPCR. **(C)** Schematic diagram of the strategy in EGR1 knockdown experiment and investigation of the viral replication state in EGR1 knockdown cells. PK-15 cells were transfected with NC siRNA or EGR1 siRNA-1819 for 48 h. The cells were infected with FMDV for 0, 12, or 16 h. Viral protein abundance was measured by Western blotting. Relative fold-change in abundance of VP1 protein was determined by densitometric analysis using Quantity One software (Bio- Rad) and normalized to β-actin. **(D)** Viral RNA levels in FMDV-infected NC siRNA or EGR1 siRNA-1819 cells at 0, 12, and 16 hpi were measured by qPCR. Viral yields in FMDV-infected NC siRNA or EGR1 siRNA-1819 cells at 16 hpi were measured by TCID_50_ assay. ^∗^*P* < 0.05 was considered as statistically significant and ^∗∗^*P* < 0.01 was considered as highly significant.

To investigate the potential role of EGR1 during FMDV infection, we evaluated the viral replication level in EGR1 overexpressed cells. PK-15 cells were transfected with different doses of EGR1 expressing plasmids, the cells were incubated with equal amounts of FMDV (0.5 MOI) at 24 h post-transfection (hpt). The viral protein and viral RNA expression level was measured at 12 hpi. Overexpression of EGR1 significantly suppressed both the viral protein expression and viral RNA replication. The viral titers in both vector and EGR1 plasmids (2 μg) transfected cells were measured and compared, which showed that FMDV yields were also decreased by overexpression of EGR1 (**Figure 2A**). To further confirm the antiviral role of EGR1 during FMDV infection, the siRNAs that target EGR1 were designed and evaluated. PK-15 cells were transfected with the NC siRNA or EGR1 siRNA for 48 h, the interference efficacy of the siRNAs was determined by qPCR analysis. The EGR1 siRNA-1819 showed the highest efficacy and was used for EGR1 knockdown assay (**Figure 2B**). PK-15 cells were transfected with EGR1 siRNA-1819, the cells were infected with FMDV at 48 hpt and incubated for another 12 or 16 h. The expression of EGR1 and FMDV VP1 protein was detected using Western blotting. Knockdown of EGR1 considerably increased VP1 protein expression during FMDV infection (**Figure 2C**). The relative fold-change in abundance of FMDV VP1 protein in FMDV-infected NC siRNA or EGR1 siRNA cells was determined by densitometric analysis and normalized to β-actin, which confirmed that knockdown of EGR1 enhanced FMDV VP1 protein expression (**Figure 2C**, right panel). Viral RNA detection also suggested that knockdown of EGR1 promoted viral replication (**Figure 2D**). The viral titers were subsequently measured at 16 hpi, which showed that knockdown of EGR1 significantly promoted FMDV propagation (**Figure 2D**, right panel). These results suggest the antiviral role of EGR1 against FMDV.

### EGR1 Enhances Type I IFN Signaling

**FIGURE 3:**
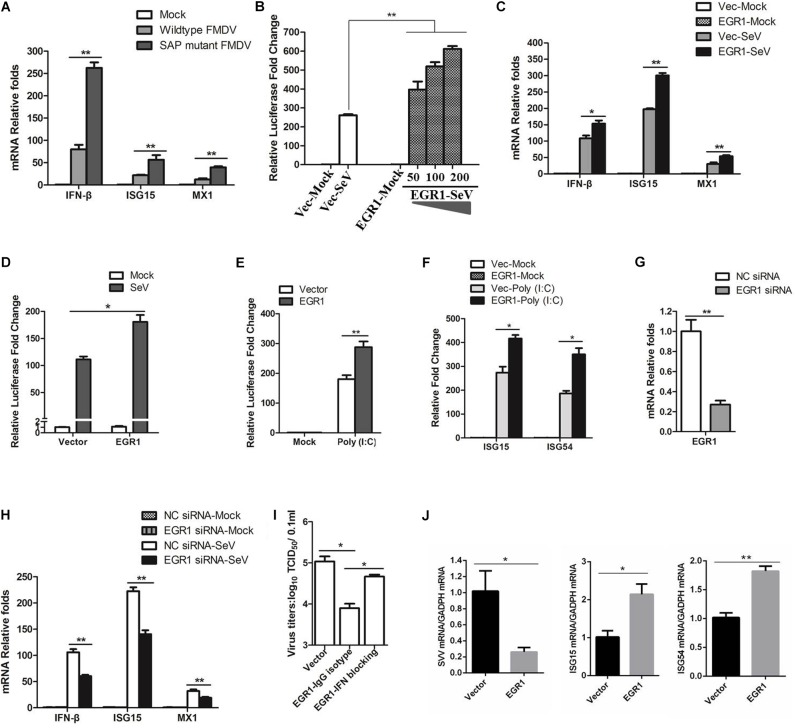
Effect of EGR1 on type I IFN signaling. **(A)** PK-15 cells were mock-infected or infected with equal amounts of SAP mutant FMDV or wildtype FMDV for 12 h. The transcripts of IFN-β, ISG15 and MX1 were detected by qPCR. **(B)** HEK293T cells were transfected with vector plasmids or increasing amounts of Myc-EGR1 plasmids (50, 100, or 200 ng) together with ISRE luciferase reporter plasmid and the internal control plasmid PRL-TK. The transfected cells were infected with SeV at 24 hpt and incubated for 16 h. Dual luciferase assay was then performed according to the Promega Dual-Luciferase Reporter Assay System protocol. **(C)** HEK293T cells were transfected with vector plasmids or Myc-EGR1 plasmids for 24 h. The transfected cells were mock-infected or infected with SeV for 16 h. The transcripts of IFN-β, ISG15 and MX1 were detected by qPCR. **(D)** HEK293T cells were transfected with vector or Myc-EGR1 plasmids together with NF-κB luciferase reporter plasmid and PRL-TK. The transfected cells were infected with SeV, and the luciferase activity was measured by dual luciferase assay. **(E)** HEK293T cells were transfected with vector plasmids or Myc-EGR1 plasmids and solvent control or Poly (I:C) together with ISRE luciferase reporter plasmid and PRL-TK. The luciferase activity was measured by dual luciferase assay. **(F)** HEK293T cells were co-transfected with vector plasmids or Myc-EGR1 plasmids and solvent control or Poly (I:C), the expression of ISG15 and ISG54 expression was measured by qPCR. **(G)** HEK293T cells were transfected with NC or EGR1 siRNA-1819 for 48 h. The EGR1 transcripts were measured by qPCR. **(H)** HEK293T cells were transfected with NC siRNA or EGR1 siRNA-1819 for 48 h. The cells were then mock-infected or infected with SeV for 16 h. The transcripts of IFN-β, ISG15 and MX1 were detected by qPCR. **(I)** PK-15 cells were transfected with equal amounts of vector or EGR1 expressing plasmids for 24 h, the EGR1-transfected cell were mock-treated or treated with IFN antibodies for 1 h and then infected with FMDV. The viral yields were measured by TCID_50_ assay at 12 hpi. **(J)** PK-15 cells were transfected with vector plasmids or Myc-EGR1 plasmids for 24 h. The transfected cells were infected with SVV for 12 h. The viral RNA, ISG15 and ISG54 expression levels were detected by qPCR. ^∗^*P* < 0.05 was considered as statistically significant and ^∗∗^*P* < 0.01 was considered as highly significant.

Both wildtype and SAP mutant FMDV infection resulted in EGR1 upregulation, however, SAP mutant FMDV resulted in a higher upregulation of EGR1 (**Figure 1A**). Previous study indicates SAP mutant FMDV infection induces higher expression of IFN-β and ISGs than wildtype FMDV infection (de los Santos et al., 2009). We also investigated the expression state of IFN-β and ISGs (ISG15 and MX1) in the cells infected by wildtype or SAP mutant FMDV. At 12 hpi, there was ∼3-fold difference in IFN-β transcripts for SAP mutant FMDV-infected cells relative to wildtype FMDV-infected cells (**Figure 3A**). A similar pattern was observed for ISG15 and MX1, varying from 2- to 4-fold higher for SAP mutant FMDV compared to wildtype FMDV (**Figure 3A**). These results were similar to the previous results reported by de los Santos et al. (2009). This showed that both EGR1 expression and type I IFN signaling were enhanced in SAP mutant FMDV-infected cells. This implied a link between EGR1 and type I IFN pathway.

To identify the role of EGR1 on type I IFN signaling, EGR1 is overexpressed in HEK293T cells, and the SeV that is routinely used to induce type I IFNs in cell culture was used to activate type I IFN signaling. Overexpression of EGR1 significantly promoted SeV-induced type I IFN signaling, showing a dose-dependent manner (**Figure 3B**). The expression of IFN-β, ISG15 and MX1 in EGR1 overexpressed cells were subsequently evaluated. The results showed that overexpression of EGR1 considerably promoted SeV-induced IFN-β, ISG15 and MX1 expression (**Figure 3C**). The effect of EGR1 on SeV-induced NF-κB activation was also evaluated by dual luciferase reporter assay, which also showed that EGR1 positively enhanced NF-κB-mediated transcriptional activity (**Figure 3D**). The role of EGR1 on Poly (I:C)-induced type I IFN signaling was further evaluated. Overexpression of EGR1 significantly promoted Poly (I:C)-induced type I IFN signaling (**Figure 3E**). The expression of Poly (I:C)-induced ISGs was also measured. The results showed that overexpression of EGR1 considerably promoted Poly (I:C)-induced ISG15 and ISG54 expression (**Figure 3F**). SeV-induced ISGs expression levels in EGR1 knockdown cells were also analyzed. The siRNA interference efficacy was also verified in HEK293T cells (**Figure 3G**). EGR1 was knocked down by transfection of siRNA, and the cells were infected by SeV at 48 hpt and incubated for 16 h. The expression of IFN-β, ISG15 and MX1 were measured. The transcript levels of IFN-β, ISG15 and MX1 remarkably decreased in EGR1 knockdown cells comparing with that in NC siRNA cell (**Figure 3H**). These results suggested a positive regulatory role of EGR1 on type I IFN signaling.

The type I IFN blocking antibody experiments were also performed. Anti-IFN-β and anti-IFN-α antibodies (5000 NU/ml) was used to block type I IFN signaling in PK-15 cells, the EGR1-overexpressed cells were treated with IFN antibodies for 1 h and then infected with FMDV. The viral yields were measured at 12 hpi. Type I IFN blocking antibodies obviously abrogated the inhibitory effects of EGR1 on FMDV propagation (**Figure 3I**). These results suggested that EGR1 suppressed FMDV replication by enhancing type I IFN signaling. We also evaluated the antiviral role of EGR1 against another picornavirus Seneca Valley Virus (SVV) which showed a close relationship with FMDV, and we found that upregulation of EGR1 also enhanced ISGs expression (ISG15 and ISG54) and suppressed SVV replication (**Figure 3J**).

### EGR1 Enhanced the Activation of the ISRE Luciferase Reporter Stimulated by TBK1 or Its Upstream Molecules (RIG-I, MDA5 and VISA)

**FIGURE 4:**
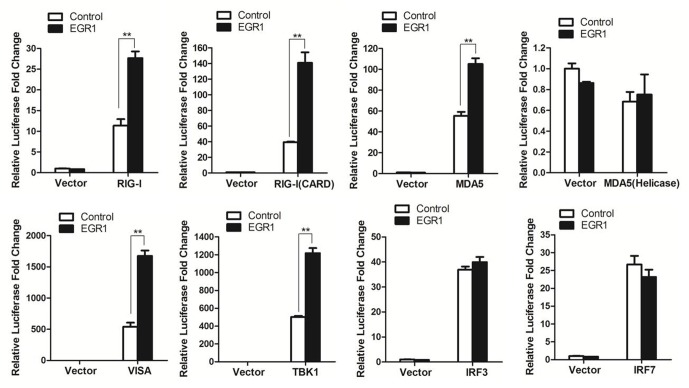
The target of EGR1 in type I IFN pathway activation. HEK293T cells were co-transfected with Myc-EGR1 or empty vector plasmids and the constructs expressing RIG-I, RIG-I(CARD), MDA5, VISA, TBK1, IRF3 or IRF7, together with ISRE luciferase reporter plasmid and the internal control plasmid PRL-TK. Dual luciferase activity was determined at 24 hpt. ^∗∗^*P* < 0.01 was considered as highly significant.

EGR1 enhanced type I IFN signaling. This raised the possibility that EGR1 targeted one or several adaptor proteins of the type I IFN signaling pathway. To screen the potential proteins that were targeted by EGR1, HEK293T cells were co-transfected with the Myc-Vector or Myc-tagged EGR1 plasmids and the indicated plasmids expressing RIG-I, RIG-I(CARD) (the CARD domain of RIG-I), MDA5(Helicase) (the Helicase domain of MDA5), VISA, TBK1, IRF3 and IRF7, together with ISRE luciferase reporter plasmid and the internal control plasmid PRL-TK. Luciferase activity was measured at 24 h after transfection. Overexpression of adaptor proteins RIG-I, RIG-I(CARD), MDA5), VISA, TBK1, IRF3 or IRF7 all activated the ISRE luciferase reporter system, and overexpression of MDA5(Helicase) did not activate the ISRE luciferase reporter system (**Figure 4**). TBK1 or its upstream proteins (RIG-I, MDA5 and VISA) mediated type I IFN signaling was significantly enhanced by overexpression of EGR1. However, overexpression of EGR1 did not promote IRF3 and IRF7 mediated type I IFN signaling (**Figure 4**). IRF3 and IRF7 are the downstream proteins of TBK1. Therefore, we speculated that TBK1 or its upstream molecules (RIG-I, MDA5 and VISA) were the target/targets of EGR1 to enhance type I IFN signal transduction.

### EGR1 Enhances Type I IFN Signaling During FMDV Infection

**FIGURE 5:**
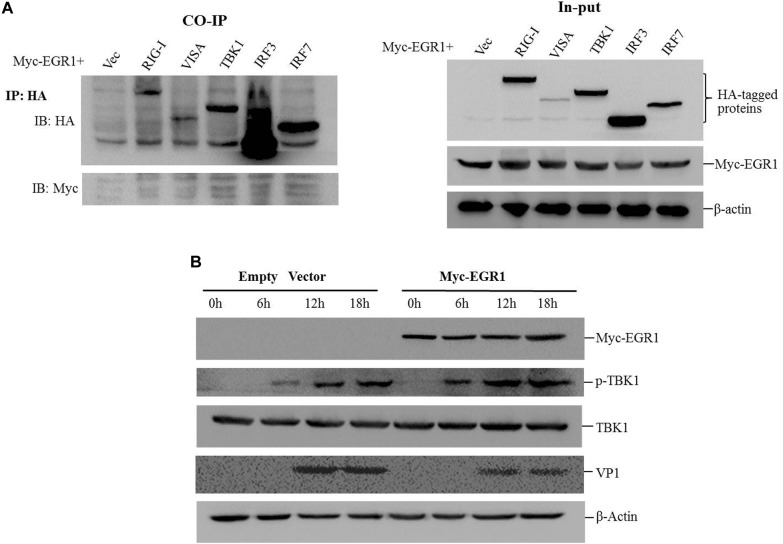
EGR1 enhances type I IFN pathway activation during FMDV infection. **(A)** HEK293T cells were co-transfected with Myc-EGR1 and HA-RIG-I, HA-VISA, HA-TBK1, HA-IRF3 or HA-IRF7 for 36 h. The transfects were immunoprecipitated with anti-HA antibodies and subjected to Western blotting analysis. Input represents the whole cell lysates. **(B)** PK-15 cells were transfected with 2 μg of empty vector or Myc-EGR1 expressing plasmids. The cells were infected with equal amounts of FMDV at 24 hpt. The infected cells were collected at 0, 6, 12, or 18 hpi and subjected to Western blotting analysis. The expression of Myc-EGR1, p-TBK1 (phosphorylated TBK1), TBK1, VP1 and β-Actin were detected.

To investigate whether EGR1 interacted with the adaptors of type I IFN pathway, the coimmunoprecipitation assay was performed by co-transfection of the Myc-EGR1 plasmids and the plasmids expressing various HA-tagged adaptors of type I IFN pathway. The transfectants were immunoprecipitated with anti-HA antibodies and subjected to Western blotting analysis. No interaction was observed between EGR1 and the adaptors (**Figure 5A**). EGR1 enhanced the activation of ISRE luciferase reporter stimulated by TBK1 and its upstream molecules. TBK1 might be a key adaptor to enhance type I IFN signaling. The influence of EGR1 on TBK1 expression and TBK1 phosphorylation levels were evaluated in FMDV-infected cells. PK-15 cells were transfected with 2 μg of Myc-EGR1 or its empty vector plasmids. The cells were infected with equal amounts of FMDV at 24 hpt and collected at 0, 6, 12, or 18 hpi. Overexpression of EGR1 had no influence on TBK1 expression during FMDV infection. However, it significantly promoted TBK1 phosphorylation levels after FMDV infection comparing with that in the empty vector transfected cells (**Figure 5B**). The VP1 was used as an indicator of viral replication. Overexpression of EGR1 also resulted in decreased VP1 abundance (**Figure 5B**). This confirmed that upregulation of EGR1 enhanced type I IFN signaling and suppressed FMDV replication.

### EGR1 Inhibits FMDV Replication Independent of Its Transcriptional Activity

**FIGURE 6:**
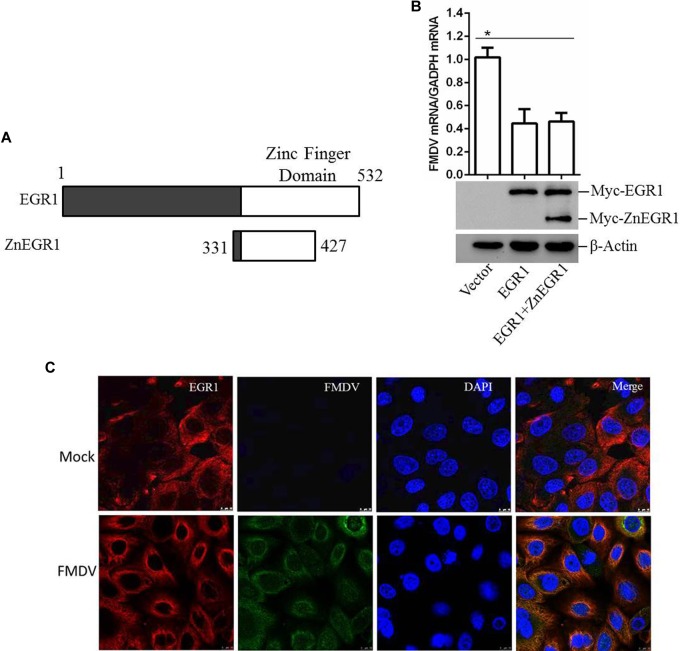
EGR1 suppresses FMDV replication independent of its transcriptional activity. **(A)** Schematic diagram of the wildtype EGR1 and ZnEGR1. **(B)** PK-15 cells were transfected with vector, Myc-EGR1 plasmids or cotransfected with Myc-EGR1 and Myc-ZnEGR1 plasmids and subjected to FMDV infection. The expression of Myc-EGR1 and Myc-ZnEGR1 was detected by Western blotting. FMDV RNA was detected by qPCR. **(C)** PK-15 cells were mock-infected or infected with FMDV for 12 h; the localization of EGR1 was detected by indirect fluorescence assay. ^∗^*P* < 0.05 was considered as statistically significant.

As a transcription factor, the transcriptional activity is significantly involved in the regulatory function of EGR1. To investigate whether the transcriptional activity is related to the antiviral function of EGR1, ZnEgr1, a previous reported dominant-negative mutant of EGR1 that lacks a transcriptional function, was used as an inhibitor of the transcriptional activity of EGR1 (Levkovitz and Baraban, 2001). The Myc-tagged ZnEGR1 expressing plasmid was constructed (**Figure 6A**). PK-15 cells were transfected with vector, Myc-EGR1 plasmids or cotransfected with Myc-EGR1 and Myc-ZnEGR1 plasmids and subjected to FMDV infection. Overexpression of EGR1 suppressed FMDV replication, and EGR1-mediated antiviral effect was not blocked by cotransfection with ZnEgr1 (**Figure 6B**). We further evaluated the localization of EGR1 in mock- or FMDV-infected cells, and we found FMDV infection did not change the localization of EGR1 compared with that in the mock-infected cells (**Figure 6C**). These data suggest that the transcriptional activity is not involved in the antiviral function of EGR1.

## Discussion

EGR1, as a multifunctional transcription factor, plays regulatory roles in a variety of cellular responses. In addition, EGR1 shows an anti-tumor function. Overexpression of EGR1 decreases tumorigenesis in nude mice and various of human tumor cell lines (Huang et al., 1994, 1995). Induction of TGFβ1 and p53 may lead to the tumor suppressor property of EGR1 (Baron et al., 2006). p53, as a tumor suppressor, has also been implicated in other functions that play important roles in disease and health (Fuhrman et al., 2009). p53-dependent antiviral defense has been widely reported (Takaoka et al., 2003; Shin-Ya et al., 2005; Muñoz-Fontela et al., 2008). Such as, p53 serves as an antiviral protein during influenza A virus infection by enhancing host innate and adaptive immune responses (Muñoz-Fontela et al., 2011). EGR1 directly induces the transcription of p53 (Liu et al., 2004), whether EGR1 is also involved in host antiviral responses remains unknown. In this study, we determined that EGR1 revealed an antiviral function against FMDV, which indicated that EGR1 is implicated in host antiviral response.

EGR1 can be upregulated upon viral infection by Epstein-Barr virus, mouse hepatitis virus (MHV), VEEV, EV71, rabies viruses and Japanese encephalitis virus infections (Saha and Rangarajan, 2003; Cai et al., 2006; Kim et al., 2013; Song et al., 2015; Baer et al., 2016). However, EGR1 expression is related to viral pathogenesis during VEEV, MHV and EV71 replication. All these studies were performed using mouse or human cells, and most of these viruses can cause central nervous system (CNS) diseases. In this study we investigated the function of porcine EGR1 and showed the antiviral role of porcine EGR1 against FMDV. FMDV infection does not cause any CNS disease. Whether the difference of species or tissue tropism resulted in the different role of EGR1 in different virus infections remain unknown. However, EGR1 has been suggested to participate in IFN-γ-STAT1 pathway in T cells (Shin et al., 2009). T-bet is a Th1-specific transcription factor that is directly involved in T cells differentiation (Djuretic et al., 2007). EGR1 regulates T-bet expression by binding to the promoter of T-bet and induces T-bet transcription (Shin et al., 2009). T-bet plays a vital role in innate immunity, and lacking of T-bet expression increases host susceptibility to inflammatory disease (Garrett et al., 2007). This implies a regulatory role of EGR1 in innate immunity. Besides, overexpression of EGR1 downregulates NFκB inhibitor (Kim et al., 2013), which also implies a potential role of EGR1 in innate immunity.

In this study, we determined that EGR1 is implicated in innate immunity during FMDV infection. A higher EGR1 expression was observed in SAP mutant FMDV-infected cells comparing with that in the wildtype FMDV-infected cells. It has been determined that SAP mutant FMDV infection resulted in stronger type I IFN signaling than wildtype FMDV infection (de los Santos et al., 2009). We found that SAP mutant FMDV infection triggered higher expression of IFN-β and ISGs than wildtype FMDV infection. This result was similar as the result reported by de los Santos et al. (2009) previously. Whether the higher expression of EGR1 correlated with the higher expression of IFN-β and ISGs was therefore investigated. Overexpression of EGR1 significantly activated type I IFN signaling and IFN-β and ISGs expression. Knockdown of EGR1 considerably impaired SeV-induced IFN-β and ISGs expression. Type I IFN blocking antibodies obviously abrogated the inhibitory effects of EGR1 on FMDV propagation. This suggested EGR1 is implicated in type I IFN pathway activation. A link between EGR1 and type I IFN pathway was reported for the first time.

Further investigation of EGR1-mediated enhancive effect showed that EGR1 promoted activation of the type I IFN signaling during FMDV infection. Overexpression of EGR1 upregulated TBK1 phosphorylation during FMDV infection. TBK1 phosphorylation enhanced type I IFN signaling and strengthened antiviral activity which subsequently suppressed FMDV replication. EGR1 is a transcription factor; however, it does not induce TBK1 expression. The interaction between EGR1 and various adaptors of type I IFN signaling pathway was not observed by performing coimmunoprecipitation assay. The role of the transcriptional activity of EGR1 for its antiviral function against FMDV was also evaluated. Suppression of the transcriptional activity of EGR1 did not affect its antiviral effect. EGR1 might enhance type I IFN signaling independent of its transcriptional activity. How does EGR1 promote TBK1 phosphorylation is not clear. Several phosphatases have been identified as regulator of phosphorylation of TBK1 (Gabhann et al., 2010; Zhao, 2013). The regulation of EGR1 on these phosphatases should be studied in future, and the detailed mechanism of EGR1 to promote TBK1 phosphorylation should be further investigated. In addition, the effect of EGR1 on the adaption of other upstream molecules of TBK1 should also be exploited.

## Conclusion

In summary, we present the first investigation of EGR1 in regulation of type I IFN signaling during FMDV infection. We determined that EGR1 showed an antiviral function against FMDV. EGR1 promoted activation of the type I IFN signaling during FMDV infection and resulted in the decreased replication of FMDV. EGR1 suppressed FMDV replication independent of its transcriptional activity. These findings identify an important role of EGR1 in enhancement of type I IFN signaling during FMDV infection. However, the exact mechanism for EGR1 to promote type I IFN signaling should be investigated in future to gain deeper understanding of EGR1-mediated functions.

## Author Contributions

ZZ, XD, and HZ conceived the study and wrote the manuscript. ZZ, XD, PL, XZ, FY, and WC performed the experiments. HT, KZ, and XL collected the data, analyzed the data, and revised the manuscript.

## Conflict of Interest Statement

The authors declare that the research was conducted in the absence of any commercial or financial relationships that could be construed as a potential conflict of interest.

## References

[B1] Baer A. Lundberg L. Swales D. Waybright N. Pinkham C. Dinman J. D. (2016). Venezuelan *Equine encephalitis* virus induces apoptosis through the unfolded protein response activation of EGR1. *J. Virol.* 90 3558–3572. 10.1128/JVI.02827-15 26792742 PMC4794670

[B2] Baron V. Adamson E. D. Calogero A. Ragona G. Dan M. (2006). The transcription factor Egr1 is a direct regulator of multiple tumor suppressors including TGFβ1, PTEN, p53 and fibronectin: Egr1 is a potential target of gene therapy for prostate cancer. *Cancer Gene Ther.* 13 115–124. 10.1038/sj.cgt.7700896 16138117 PMC2455793

[B3] Barrientos T. Frank D. Kuwahara K. Bezprozvannaya S. Pipes G. C. Bassel-Duby R. (2007). Two novel members of the ABLIM protein family, ABLIM-2 and -3, associate with stars and directly bind F-actin. *J. Biol. Chem.* 282 8393–8403. 10.1074/jbc.M607549200 17194709

[B4] Bouguedour R. Ripani A. (2016). Review of the foot and mouth disease situation in North Africa and the risk of introducing the disease into Europe. *Rev. Sci. Tech.* 35 757–768. 10.20506/rst.35.3.2566 28332653

[B5] Brand T. Sharma H. S. Fleischmann K. E. Duncker D. J. Mcfalls E. O. Verdouw P. D. (1992). Proto-oncogene expression in porcine myocardium subjected to ischemia and reperfusion. *Circ. Res.* 71 1351–1360. 10.1161/01.RES.71.6.1351 1385005

[B6] Cai Y. Liu Y. Zhang X. (2006). Induction of transcription factor Egr-1 gene expression in astrocytoma cells by *Murine coronavirus* infection. *Virology* 355 152–163. 10.1016/j.virol.2006.07.012 16908043 PMC1851928

[B7] Cubero F. J. Nieto N. (2012). Arachidonic acid stimulates TNFα production in kupffer cells via a reactive oxygen species-pERK1/2-Egr1-dependent mechanism. *Am. J. Physiol. Gastrointest. Liver Physiol.* 303 228–239. 10.1152/ajpgi.00465.2011 22538404 PMC3404567

[B8] de los Santos T. Diaz-San Segundo F. Zhu J. Koster M. Dias C. C. Grubman M. J. (2009). A conserved domain in the leader proteinase of foot-and-mouth disease virus is required for proper subcellular localization and function. *J. Virol.* 83 1800–1810. 10.1128/JVI.02112-08 19052079 PMC2643771

[B9] Diaz-San Segundo F. Weiss M. Perez-Martin E. Dias C. C. Grubman M. J. De Los Santos T. (2012). Inoculation of swine with foot-and-mouth disease SAP-mutant virus induces early protection against disease. *J. Virol.* 86 1316–1327. 10.1128/JVI.05941-11 22114339 PMC3264347

[B10] Djuretic I. M. Levanon D. Negreanu V. Groner Y. Rao A. Ansel K. M. (2007). Transcription factors T-bet and Runx3 cooperate to activate Ifng and silence Il4 in T helper type 1 cells. *Nat. Immunol.* 8 145–153. 10.1038/ni1424 17195845

[B11] Domingo E. Pariente N. Airaksinen A. Gonzalez-Lopez C. Sierra S. Herrera M. (2005). Foot-and-mouth disease virus evolution: exploring pathways towards virus extinction. *Curr. Top. Microbiol. Immunol.* 288 149–173. 15648178 10.1007/3-540-27109-0_7 PMC7121672

[B12] Fuhrman L. E. Goel A. K. Smith J. Shianna K. V. Aballay A. (2009). Nucleolar proteins suppress *Caenorhabditis elegans* innate immunity by inhibiting p53/CEP-1. *PLoS Genet.* 5:e1000657. 10.1371/journal.pgen.1000657 19763173 PMC2734340

[B13] Gabhann J. N. Higgs R. Brennan K. Thomas W. Damen J. E. Ben Larbi N. (2010). Absence of SHIP-1 results in constitutive phosphorylation of tank-binding kinase 1 and enhanced TLR3-dependent IFN-beta production. *J. Immunol.* 184 2314–2320. 10.4049/jimmunol.0902589 20100929

[B14] Garrett W. S. Lord G. M. Punit S. Lugo-Villarino G. Mazmanian S. K. Ito S. (2007). Communicable ulcerative colitis induced by T-bet deficiency in the innate immune system. *Cell* 131 33–45. 10.1016/j.cell.2007.08.017 17923086 PMC2169385

[B15] Grubman M. J. Baxt B. (2004). Foot-and-mouth disease. *Clin. Microbiol. Rev.* 17 465–493. 10.1128/CMR.17.2.465-493.2004 15084510 PMC387408

[B16] Huang R. P. Darland T. Okamura D. Mercola D. Adamson E. D. (1994). Suppression of v-sis-dependent transformation by the transcription factor, Egr-1. *Oncogene* 9 1367–1377. 8152797

[B17] Huang R. P. Liu C. Fan Y. Mercola D. Adamson E. D. (1995). Egr-1 negatively regulates human tumor cell growth via the DNA-binding domain. *Cancer Res.* 55 5054–5062. 7585551

[B18] Jo G. Shin S. Y. Lee Y. Hyun J. Dong K. S. Park J. C. (2011). A compound isolated from *Rumex japonicus* induces early growth response gene-I expression. *J. Korean Soc. Appl. Biol. Chem.* 54 637–643.

[B19] Khachigian L. M. Anderson K. R. Halnon N. J. Gimbrone M. A. Resnick N. Collins T. (1997). Egr-1 is activated in endothelial cells exposed to fluid shear stress and interacts with a novel shear-stress-response element in the PDGF a-chain promoter. *Arterioscler. Thromb. Vasc. Biol.* 17 2280–2286. 10.1161/01.ATV.17.10.2280 9351401

[B20] Kim J. H. Kim W. S. Park C. (2013). *Epsteinâ-Barr virus* latent membrane protein 1 increases genomic instability through Egr-1-mediated up-regulation of activation-induced cytidine deaminase in B-cell lymphoma. *Leuk. Lymphoma* 54 2035–2040. 10.3109/10428194.2013.769218 23363221

[B21] Levkovitz Y. Baraban J. M. (2001). A dominant negative inhibitor of the Egr family of transcription regulatory factors suppresses cerebellar granule cell apoptosis by blocking c-Jun activation. *J. Neurosci.* 21 5893–5901. 10.1523/JNEUROSCI.21-16-05893.2001 11487612 PMC6763154

[B22] Li D. Yang W. Yang F. Liu H. Zhu Z. Lian K. (2016). The VP3 structural protein of foot-and-mouth disease virus inhibits the IFN-β signaling pathway. *FASEB J.* 30 1757–1766. 10.1096/fj.15-281410 26813975

[B23] Li W. Zhu Z. Cao W. Yang F. Zhang X. Li D. (2016). Esterase D enhances type I interferon signal transduction to suppress foot-and-mouth disease virus replication. *Mol. Immunol.* 75 112–121. 10.1016/j.molimm.2016.05.016 27267271

[B24] Li D. P. Ilnytskyy Y. Kovalchuk A. Khachigian L. M. Bronson R. T. Wang B. (2013). Crucial role for early growth response-1 in the transcriptional regulation of miR-20b in breast cancer. *Oncotarget* 4 1373–1387. 10.18632/oncotarget.1165 23945289 PMC3824527

[B25] Liu J. Grogan L. Nau M. M. Allegra C. J. Chu E. Wright J. J. (2004). Physical interaction between p53 and primary response gene Egr-1. *Henan Med. Res.* 18 863–870. 11251186 10.3892/ijo.18.4.863

[B26] Mason P. W. Grubman M. J. Baxt B. (2003). Molecular basis of pathogenesis of FMDV. *Virus Res.* 91 9–32. 10.1016/S0168-1702(02)00257-5 12527435

[B27] McMahon S. B. Monroe J. G. (1996). The role of early growth response gene 1 (egr-1) in regulation of the immune response. *J. Leukoc. Biol.* 60 159–166. 10.1002/jlb.60.2.159 8773576

[B28] Muñoz-Fontela C. Macip S. Martínez-Sobrido L. Brown L. Ashour J. García-Sastre A. (2008). Transcriptional role of p53 in interferon-mediated antiviral immunity. *J. Exp. Med.* 205 1929–1938. 10.1084/jem.20080383 18663127 PMC2525597

[B29] Muñoz-Fontela C. Pazos M. Delgado I. Murk W. Mungamuri S. K. Lee S. W. (2011). p53 serves as a host antiviral factor that enhances innate and adaptive immune responses to influenza A virus. *J. Immunol.* 187 6428–6436. 10.4049/jimmunol.1101459 22105999 PMC3275346

[B30] Nishi H. Nishi K. H. Johnson A. C. (2002). Early growth response-1 gene mediates up-regulation of epidermal growth factor receptor expression during hypoxia. *Cancer Res.* 62 827–834. 11830539

[B31] Pagel J. I. Deindl E. (2011). Early growth response 1–a transcription factor in the crossfire of signal transduction cascades. *Indian J. Biochem. Biophys.* 48 226–235. 22053691

[B32] Papanikolaou N. A. Tillinger A. Liu X. Papavassiliou A. G. Sabban E. L. (2014). A systems approach identifies co-signaling molecules of early growth response 1 transcription factor in immobilization stress. *BMC Syst. Biol.* 8:100. 10.1186/s12918-014-0100-8 25217033 PMC4363937

[B33] Paton D. J. Taylor G. (2011). Developing vaccines against foot-and-mouth disease and some other exotic viral diseases of livestock. *Philos. Trans. R. Soc. B Biol. Sci.* 366 2774–2781. 10.1098/rstb.2011.0107 21893540 PMC3146785

[B34] Rodriguez Pulido M. Saiz M. (2017). Molecular mechanisms of foot-and-mouth disease virus targeting the host antiviral response. *Front. Cell. Infect. Microbiol.* 7:252. 10.3389/fcimb.2017.00252 28660175 PMC5468379

[B35] Rweyemamu M. Roeder P. Mackay D. Sumption K. Brownlie J. Leforban Y. (2008a). Epidemiological patterns of foot-and-mouth disease worldwide. *Transbound. Emerg. Dis.* 55 57–72. 10.1111/j.1865-1682.2007.01013.x 18397509

[B36] Rweyemamu M. Roeder P. Mackay D. Sumption K. Brownlie J. Leforban Y. (2008b). Planning for the progressive control of foot-and-mouth disease worldwide. *Transbound. Emerg. Dis.* 55 73–87. 10.1111/j.1865-1682.2007.01016.x 18397510

[B37] Saha S. Rangarajan P. N. (2003). Common host genes are activated in mouse brain by Japanese encephalitis and rabies viruses. *J. Gen. Virol.* 84 1729–1735. 10.1099/vir.0.18826-0 12810866

[B38] Shin H. J. Lee J. B. Park S. H. Chang J. Lee C. W. (2009). T-bet expression is regulated by EGR1-mediated signaling in activated T cells. *Clin. Immunol.* 131 385–394. 10.1016/j.clim.2009.02.009 19307156

[B39] Shin-Ya M. Hirai H. Satoh E. Kishida T. Asada H. Aoki F. (2005). Intracellular interferon triggers jak/stat signaling cascade and induces p53-dependent antiviral protection. *Biochem. Biophys. Res. Commun.* 329 1139–1146. 10.1016/j.bbrc.2005.02.088 15752772

[B40] Skerka C. Decker E. L. Zipfel P. F. (1995). A regulatory element in the human interleukin 2 gene promoter is a binding site for the zinc finger proteins Sp1 and EGR-1. *J. Biol. Chem.* 270 22500–22506. 10.1074/jbc.270.38.22500 7673240

[B41] Sobrino F. Domingo E. (2001). Foot-and-mouth disease in Europe. FMD is economically the most important disease of farm animals. Its re-emergence in Europe is likely to have consequences that go beyond severe alterations of livestock production and trade. *EMBO Rep.* 2 459–461. 10.1093/embo-reports/kve122 11415972 PMC1083915

[B42] Song Y. Cheng X. Yang X. Zhao R. Wang P. Han Y. (2015). Early growth response-1 facilitates enterovirus 71 replication by direct binding to the viral genome RNA. *Int. J. Biochem. Cell Biol.* 62 36–46. 10.1016/j.biocel.2015.02.012 25724735

[B43] Takaoka A. Hayakawa S. Yanai H. Stoiber D. Negishi H. Kikuchi H. (2003). Integration of interferon-|α|/|β| signalling to p53 responses in tumour suppression and antiviral defence. *Nature* 424 516–523. 10.1038/nature01850 12872134

[B44] Trottier C. Colombo M. Mann K. K. Miller W. H. Jr. Ward B. J. (2009). Retinoids inhibit measles virus through a type I IFN-dependent bystander effect. *FASEB J.* 23 3203–3212. 10.1096/fj.09-129288 19447880

[B45] Zai-Xin L. (2015). Progress and prospect of the technologies to control foot-and-mouth disease and its pathogen characteristics worldwide. *Sci. Agric. Sin.* 48 3547–3564.

[B46] Zhao W. (2013). Negative regulation of TBK1-mediated antiviral immunity. *FEBS Lett.* 587 542–548. 10.1016/j.febslet.2013.01.052 23395611 PMC7094513

[B47] Zheng H. X. He J. J. Guo J. H. Jin Y. Yang F. Lv L. (2012). Genetic characterization of a new pandemic Southeast Asia topotype strain of serotype O foot-and-mouth disease virus isolated in China during 2010. *Virus Genes* 44 80–88. 10.1007/s11262-011-0670-0 21932049

[B48] Zhou Q. Lin H. Wang S. Wang S. Ran Y. Liu Y. (2014). The ER-associated protein ZDHHC1 is a positive regulator of DNA virus-triggered, MITA/STING-dependent innate immune signaling. *Cell Host Microbe* 16 450–461. 10.1016/j.chom.2014.09.006 25299331

[B49] Zhu Z. Shi Z. Yan W. Wei J. Shao D. Deng X. (2013). Nonstructural protein 1 of influenza A virus interacts with human guanylate-binding protein 1 to antagonize antiviral activity. *PLoS One* 8:e55920. 10.1371/journal.pone.0055920 23405236 PMC3566120

[B50] Zhu Z. Wang G. Yang F. Cao W. Mao R. Du X. (2016). Foot-and-mouth disease virus viroporin 2B antagonizes RIG-I mediated antiviral effects by inhibition of its protein expression. *J. Virol.* 90 11106–11121. 10.1128/JVI.01310-16 27707918 PMC5126369

[B51] Zhu Z. Yang F. Zhang K. Cao W. Jin Y. Wang G. (2015). Comparative proteomic analysis of wild-type and SAP domain mutant foot-and-mouth disease virus-infected porcine cells identifies the ubiquitin-activating enzyme UBE1 required for virus replication. *J. Proteome Res.* 14 4194–4206. 10.1021/acs.jproteome.5b00310 26354183

